# The Cross-Neutralizing Activity of Enterovirus 71 Subgenotype C4 Vaccines in Healthy Chinese Infants and Children

**DOI:** 10.1371/journal.pone.0079599

**Published:** 2013-11-19

**Authors:** Qunying Mao, Tong Cheng, Fengcai Zhu, Jingxin Li, Yiping Wang, Yanping Li, Fan Gao, Lisheng Yang, Xin Yao, Jie Shao, Ningshao Xia, Zhenglun Liang, Junzhi Wang

**Affiliations:** 1 National Institutes for Food and Drug Control, Beijing, China; 2 National Institute of Diagnostics and Vaccine Development in Infectious Disease, School of Life Science, Xiamen University, Xiamen, China; 3 Jiangsu Provincial Center for Disease Control and Prevention, Nanjing, China; 4 The Center for Disease Control and Prevention of the Guangxi Zhuang Autonomous Region, Nanning, China; Johns Hopkins School of Public Health, United States of America

## Abstract

**Background:**

EV71 is one of major etiologic causes of hand-foot-mouth disease (HFMD) and leads to severe neurological complications in young children and infants. Recently inactivated EV71 vaccines have been developed by five manufactures and clinically show good safety and immunogenicity. However, the cross-neutralizing activity of these vaccines remains unclear, and is of particular interest because RNA recombination is seen more frequently in EV71 epidemics.

**Methodology/Principal Findings:**

In this post-hoc study, sera from a subset of 119 infants and children in two clinical trials of EV71 subgenotype C4 vaccines (ClinicalTrials.gov Identifier: NCT01313715 and NCT01273246), were detected for neutralizing antibody (NTAb) titres with sera from infected patients as controls. Cytopathogenic effect method was employed to test NTAb against EV71 subgenotype B4, B5, C2, C4 and C5, which were prominent epidemic strains worldwide over the past decade. To validate the accuracy of the results, ELISpot assay was employed in parallel to detect NTAb in all the post-vaccine sera. After two-dose vaccination, 49 out of 53 participants in initially seronegative group and 52 out of 53 participants in initially seropositive group showed less than 4-fold differences in NTAb titers against five EV71 strains, whereas corresponding values among sera from pediatric patients recovering from EV71-induced HFMD and subclinically infected participants were 8/8 and 41/43, respectively. The geometric mean titers of participants against five subgenotypes EV71 all grew significantly after vaccinations, irrespective of the baseline NTAb titer. The relative fold increase in antibody titers (NTAb-FI) against B4, B5, C2, and C5 displayed a positive correlation to the NTAb-FI against C4.

**Conclusions/Significance:**

The results demonstrated broad cross-neutralizing activity induced by two C4 EV71 vaccines in healthy Chinese infants and children. However, the degree of induced cross-protective immunity, and the potential escape evolution for EV71 still need to be monitored and researched in future for these new vaccines.

## Introduction

Enterovirus 71 (EV71), a member of the *Enterovirus* genus in the *Picornaviridae* family, is a highly infectious agent that causes hand-foot-mouth disease (HFMD), herpangina, aseptic meningitis, encephalitis, and pulmonary edema in humans [1]–[5]. The past ten years have witnessed an increase in the severity of HFMD onset, the incidence of severe HFMD cases, and the number of mortalities in the West Pacific regions [6]–[9]. EV71 is now considered as the most dangerous neurotropic enterovirus of the post-polio era [8]–[9]. In order to prevent and control EV71-associated epidemics, researchers in Mainland China, Chinese Taiwan, and Singapore have developed five inactivated EV71 vaccines, employing one EV71 strain of subgenotype C4, B3 or B4, respectively [10]–[14]. Candidates which induced high NTAb titers and exert protective effects in animals have entered clinical trials [14]. The clinical trial results also suggest that these vaccines have good safety, and satisfactory immunogenicity when tested by EV71 strains which belong to the same subgenotypes with their vaccine strains [13], [15]. However, EV71 was classfied 11 subgenotypes (A, B1–B5, and C1–C5). In recent years, numerous large outbreaks of HFMD caused by different subgenotype of EV71 have occurred in Eastern and Southeastern Asian countries [3], [16]–[20]. By now, cross-protection against other genotypes and subgenotypes EV71 for EV71 vaccines in human has yet been elucidated, which is a key issue concerning the practical protective effects of the vaccine.

In our post-hoc study, serum samples from a subset of 119 participants (aged 6 months to 11 years) in two clinical trials of inactivated EV71 (subgenotype C4) vaccines (ClinicalTrials.gov Identifier: NCT01313715 and NCT01273246) [21], [22] were detected for neutralizing antibody (NTAb) titers. Cytopathogenic effect (CPE) method was employed for B4, B5, C2, C4 and C5 subgenotypes EV71 strains, which were prominent epidemic strains worldwide over the past decade [20]. Our results suggest that inactivated EV71 vaccines derived from subgenotype C4 have a broad cross-neutralizing activity in Chinese infants and children.

## Materials and Methods

### 1. Serum samples from EV71 vaccine clinical trial participants

Serum samples were acquired from a subset of 72 participants (aged 6 months to 5 years) in a clinical trial with a ClinicalTrials.gov Identifier of NCT01313715; and from 47 participants (aged from 6 months to 11 years) in a clinical trial with a ClinicalTrials.gov Identifier of NCT01273246 (Table 1) [21], [22]. Both trials also included samples from healthy participants with no HFMD history. Participants received the inactivated subgenotype C4 EV71 vaccines (vaccine A and vaccine B) on day 0 and day 28; these vaccines were developed by Sinovac Biotech Co., Ltd or Bejing Vigoo Biological Co., Ltd with different viral strains (H07 and FY7VP5/AH/CHN/2008), cell culture system (cell factories and bioreator system), production process, and vaccine dosage [15], [21], [22]. The samples were sequentially selected based on the following criteria: the participant had a day 56 post-vaccination titer against EV71 subgenotype C4 of >1∶8 [21], [22], and a serum sample (collected before vaccination and/or 28 days after two doses) residual volume greater than 1.0 ml (to be used for cross-neutralizing antibody tests). Written informed consent was received from donors' guardians.

**Table 1:** Demographic characteristics of the serum donors.

	Healthy subjects from clinical trials				EV71-associated HFMD cases
	NCT01313715		NCT01273246		
	Initially seronegative	Initially seropositive	Initially seronegative	Initially seropositive	
No.	29	43	24	23	8
Age (months)	30.5 (10.9)	43.1 (10.4)	20.3 (8.7)	57.6 (34.3)	40.9 (16.5)
Boys	18 (62.1%)	21 (48.8%)	12 (50.0%)	9 (39.1%)	3 (37.5%)
Girls	11 (37.9%)	22 (51.2%)	12 (50.0%)	14 (60.9%)	5 (62.5%)
Dosage of vaccination*					
Low	15 (51.7%)	15 (34.9%)	7 (29.2%)	2 (8.7%)	-
Middle	14 (48.3%)	13 (30.2%)	8 (33.3%)	13 (56.5%)	-
High	0 (0.0%)	15 (34.9%)	9 (37.5%)	8 (34.8%)	-

Data are shown as mean (SD) and number (%).

In trial NCT013137: Low = 160 U/0.5 ml/dose, Middle = 320 U/0.5 ml/dose, High = 640 U/0.5 ml/dose.

In trial NCT012732: Low = 100 U/0.5 ml/dose, Middle = 200 U/0.5 ml/dose, High = 400 U/0.5 ml/dose.

### 2. Serum samples from HFMD pediatric patients

In the region of Guangxi Province of China, eight EV71-infected HFMD patients were diagnosed during the outbreak of HFMD in 2010–2011. After the study was approved by the Guangxi CDC Ethics Committee, serum samples were collected from these patients 28 days after the onset of the disease, to serve as serum samples from HFMD recovery patients.

All serum samples were stored at −20°C before use.

### 3. Cells and Virus Strains

Rhabdomyosarcoma cells (RD cells: ATCC, CCL-136) were cultured in MEM solution (GIBCOL; USA) supplemented with 10% fresh bovine calf serum (GIBCOL; USA), 2 mM L-glutamine (GIBCOL; USA), 100 IU/ml penicillin and streptomycin (GIBCOL; USA). EV71 clinical isolates C2, C4, C5, B4, and B5 were kindly provided by National Institute of Diagnostics and Vaccine Development in Infectious Disease, School of Life Science, Xiamen University, China; and by Graduate Institute of Clinical Medicine, National Taiwan University and Hospital, Taiwan (Figure S1). These EV71 clinical isolates were adapted for growth in RD cells. Virus titers ranged from 10^7^ to 10^8^ TCID_50_/ml. The virus strains were proliferated in RD cells. The infected RD cells were frozen and thawed three times to release the virus. After centrifuging at 200×g for 10 min to remove cell debris, the supernatant was collected and stored at −70°C.

### 4. CPE assays for the detection of NTAb against different EV71 genotypes

Classical CPE assay was employed to measure NTAb against EV71 genotypes [11], [21], [22]. Blood samples were inactivated at 56°C for 30 minutes, serially diluted two-fold from 1∶8 and mixed with equal volumes of TCID_50_ of a EV71 strain. The mixture was dispensed into a 96-well microplate and incubated at 37°C for 2 hours. RD cells (1–2×10^5^ cells/mL) were added to the mixture. The plates were then placed in a CO_2_ incubator at 35°C for seven days. CPE was observed by microscopy. EV71 national standards were included in each test as a control for the reproducibility of the results [23]. Neutralizing antibody titers of EV71 were defined as the dilution rate showing 50% inhibition of the CPE. NTAb titers equal to or greater than 1∶8 were defined as seropositive [11], [21]–[24].

### 5. Enzyme-linked Immunospot (ELISpot) Assay for the measurement of NTAb against different EV71 genotypes

In order to validate the accuracy of our cross-protection results acquired using the CPE NTAb detection method, a microscale ELISpot assay was employed simultaneously for NTAb detection. This technique has been used previously for respiratory syncytial virus and HCMV [25], [26]. The ELISpot neutralization assay uses a monoclonal antibody against the viral capsid protein VP1 to detect EV71-infected cells. The same cells and EV71 virus strains of five subgenotypes with CPE method were used. After immunoperoxidase staining, infected cells were counted by an automated ELISpot analyzer. Neutralizing antibodies can suppress viral infection, leading to a reduction in the number of infected cells, which is reflected by a reduction in the number of spots. The neutralization titers were read as the highest dilution that completely inhibited over 50% of the number of spots. Results from the ELISpot neutralization assay were consistent with those from the classical CPE-based neutralization assay [27].

### 6. Statistical Analysis

Curve fitting and ID_50_ value determination were performed with Prism 5 software (GraphPad Software, Inc.). Seropositive rates were compared by chi-square test. Statistical analysis of the geometric mean titers (GMTs) was done by SPSS 10.0 software, after subjecting the data to a log 2 transformation. This transformation was effective in stabilizing the dispersion and rendered the variances independent of the means. If the titers of neutralizing antibodies were negative, they were assumed to be 1∶4 for calculation purposes. A paired t-test was performed, with p<0.05 considered statistically significant. Seroconversion is defined as pre-vaccination titre less than 1∶8 and post-vaccination titre 1∶32 or more, or pre-vaccination titre 1∶8 or more and at least four-fold increase post-vaccination.

## Results

### 1. Participants

Serum samples from 119 participants who received two doses of inactivated EV71 vaccine on days 0 and 28 were analyzed in this study. The demographic characteristics of these blood donors are shown in Table 1. The donors were stratified into two subgroups according to their baseline EV71 NTAb level pre-vaccination [21], [22]. The participants with baseline NTAb titer <1∶8 were categorized as the initially seroneg'ative group who were considered to be uninfected case, while the remaining participants with NTAb titer ≥1∶8 were categorized as the initially seropositive group who were considered to be subclinically infected cases because they are all children without the history of HFMD.

This study also utilized the recovery-period sera of eight EV71-infected HFMD patients (aged from 20 months to 73 months) in Guangxi Province from 2010–2011 (Table S1). These patients were categorized as the EV71 patient group (considered to be clinical infected cases, Table 1).

### 2. The NTAb response of participants after immunized with two C4 EV71 vaccines

Among 119 participants, serum samples from 83 participants were paired from pre-vaccination and 28 days after two-dose vaccination. NTAb titer of each paired serum sample was measured by CPE method with EV71 subgenotype B4, B5, C2, C4 and C5, respectively. As shown in Table 2, NTAb GMTs of the participants against 5 different subgenotypes EV71 all grew significantly after vaccination, irrespective of the baseline NTAb titers (all, p<0.0001). Among initially seronegative participants, the seroconversion rates of participants immunized with vaccine A were all 100% for 5 different subgenotypes and of participants immunized with vaccine B were 96.6–100% for 5 different subgenotypes; among initially seropositive participants who are immunized with vaccine A, the seroconversion rates were 56.7–73.3% for 5 different subgenotypes. There were no significant differences in seroconversion rate among the five EV71 subgenotypes in both initially seronegative participants and initially seropositive participants after vaccination (Table 2). The fold increases in geometric mean titers (GMFIs) were 64.0–91.6, 44.3–57.7 and 5.4–6.8 fold in above groups after vaccination. No significant differences in GMFIs among the five EV71 subgenotypes were found in all groups (Table 2). However, significant NTAb titer increases against five different genotypes were observed in initially seronegative infants and children, compared with initially seropositive infants and children (p<0.05). All these results indicated that NTAb titer induced by C4 EV71 vaccine which was tested with EV71 strains of five genotypes was similar.

**Table 2:** Post-vaccination EV71 neutralizing antibody response against EV71 strains of subgenotype B4, B5, C2, C4 and C5, stratified by pre-vaccination titer.

Groups	Vaccination	Number of participants	Subgenotypes EV71 used for testing	Pre-vaccination^a^	28 days after two vaccinations		
				GMTs (95% CI)	Seroconversion rate (95% CI)	GMTs (95% CI)	GMFI (95% CI)
Initially sero- negative(<1∶8)	Vaccine A	24	C4	4·0	100·0% (85·8–100·0)	177·3 (108·6–289·7)*	44·3 (27·1–72·4)
			B4	4·0	100·0% (85·8–100·0)	199·1 (118·5–334·4)*	49·8 (29·6–83·6)
			B5	4·0	100·0% (85·8–100·0)	182·5 (118·2–281·9)*	45·6 (29·6–70·5)
			C2	4·0	100·0% (85·8–100·0)	223·0 (144·6–365·8)*	57·5 (36·1–91·4)
			C5	4·0	100·0% (85·8–100·0)	193·4 (120·3–310·8)*	48·3 (30·1–77·7)
	Vaccine B&	29	C4	4·0	100·0% (88·1–100·0)	256·0 (156·3–419·2)*	64·0 (39·1–104·8)
			B4	4·0	100·0% (88·1–100·0)	366·4 (240·6–557·9)*	91·6 (60·2–139·5)
			B5	4·0	96·6% (82·2–99·9)	288·5 (183·7–453·2)*	72·1 (45·9–113·3)
			C2	4·0	96·6% (82·2–99·9)	262·2 (158·1–434·7)*	65·5 (39·5–108·7)
			C5	4·0	100·0% (88·1–100·0)	302·6 (189·0–484·6)*	75·7 (47·2–12·2)
Initially seropositive(≥1∶8)	Vaccine B&	30	C4	104·0 (77·2–140·0)	66·7% (47·2–82·7)#	630·5 (414·9–958·3)*	6·1 (4·1–9·0)
			B4	104·0 (81·3–133·0)	63·3% (43·9–80·1)#	660·4 (408·1–1068·5)*	6·4 (3·9–10·4)
			B5	106·4 (77·4–146·3)	56·7% (37·4–74·5)#	574·9 (367·7–898·7)*	5·4 (3·5–8·3)
			C2	106·4 (77·4–146·3)	73·3% (54·1–87·7)#	724·3 (465·3–1127·4)*	6·8 (4·6–10·1)
			C5	99·3 (74·1–133·0)	70·0% (50·6–85·3)#	602·1 (388·7–932·6)*	6·1 (4·0–9·2)

N: number of total participants included in the analyses. Data are GMT (95% CI) or GMFI (95% CI). GMT, geometric mean titer; GMFI, fold increase in geometric mean titers. Seroconversion is defined as pre-vaccination titre less than 1∶8 and post-vaccination titre 1∶32 or more, or pre-vaccination titre 1∶8 or more and at least four-fold increase post-vaccination.

a Negative antibody titers (i.e., <1∶8) were assigned a value of 1∶4 for calculation purposes.

# Seroconversion rate was significantly higher in the initially seronegative infants and children than the initially seropositive infants and children after vaccination (p<0.05 in both cases).

* The GMTs of neutralizing antibodies against five EV71 subgenotypes increase significantly after vaccination (p<0.0001 in both cases).

& Seroconversion rate were not significantly different among the five EV71 subgenotypes after vaccination. (initially seronegative group: χ^2^ = 3.00, p>0.05; initially seropositive group: χ^2^ = 2.19, p>0.05).

**Figure 1:**
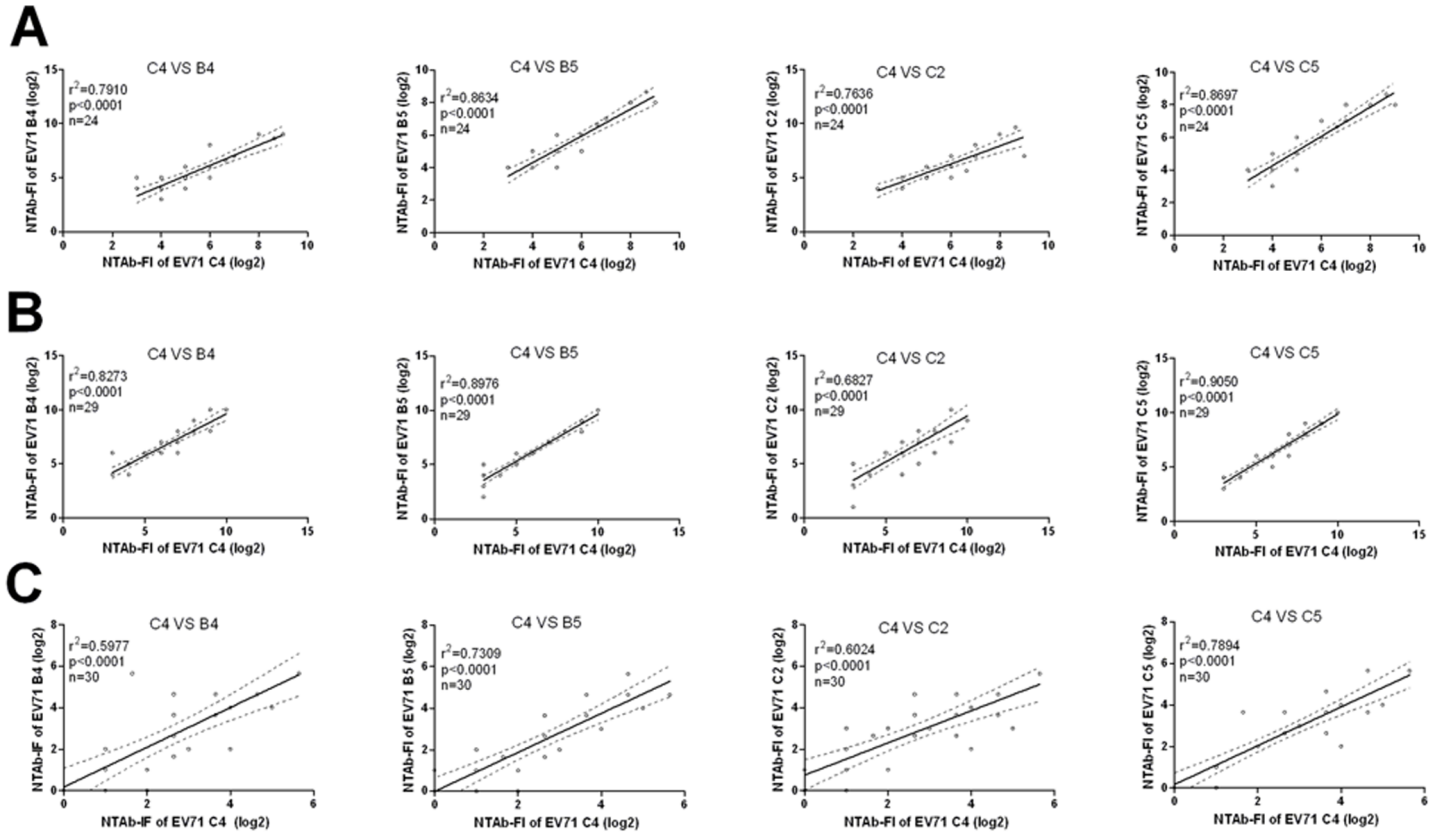
Correlation analyses of NTAb-FI against different EV71 genotypes in infants and children after vaccination. A) Correlation of NTAb-FI against different genotypes in response to Vaccine A in initially seronegative infants and children. B) Correlation of NTAb-FI against different genotypes in response to Vaccine B in initially seronegative infants and children. C) Correlation of NTAb-FI against different genotypes in response to Vaccine B in initially seropositive infants and children.

To further analyze the NTAb response against different EV71 subgenotypes induced by C4 EV71 vaccine, We made up the correlation between the fold increase of NTAb titers (NTAb-FI) against C4 subgenotype with that of the other four subgenotypes (Figure 1). The results showed that in the initially seronegative group, NTAb-FI against the other four genotypes were significantly correlated with that against C4 (p<0.0001 in both cases, r^2^ in the range of 0.7636–0.8697 and 0.6827–0.9050, respectively). Among initially seropositive infants and children, the r^2^ value was between 0.5977 and 0.7894, with p<0.0001 in both cases. The NTAb-FI against B4, B5, C2 and C5 showed a positive correlation with that against C4 (Figure 1) among children and infants vaccinated with two C4 genotype EV71 vaccines. Correlation in NTAb-FI between C5 and C4 was indicated by the r^2^ values of 0.9050, 0.8697, and 0.7894, which were the highest among other subgenotypes. These results suggested that compared to other genotypes, the antigenic properties of C5 are similar to those of C4, which is consistent with the results from EV71 VP1 evolutionary genetics analysis (Figure S1).

### 3. The cross-neutralizing effect of sero-antibodies induced by two EV71 vaccines was tested against EV71 strains of five subgenotypes

**Figure 2:**
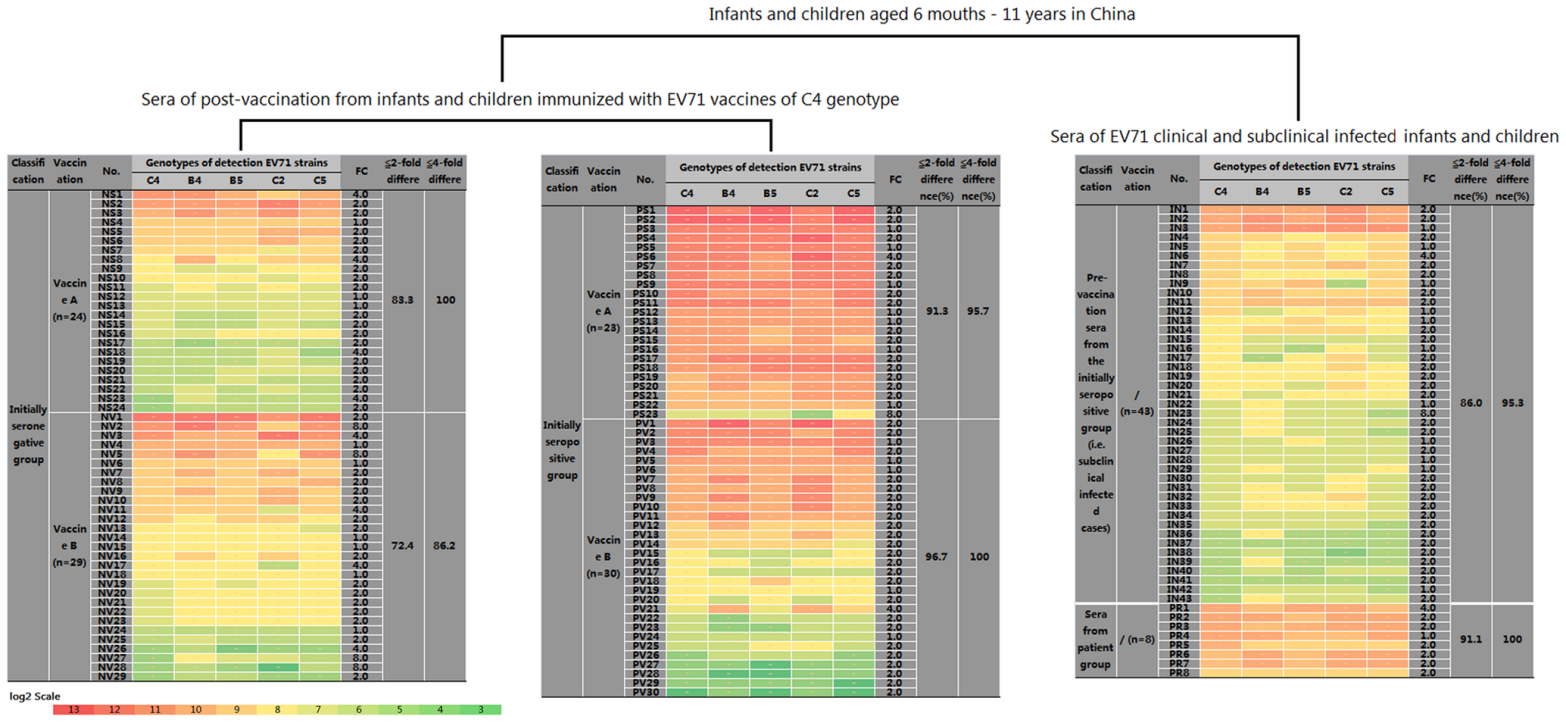
Cross-neutralizing activity of the C4 EV71 inactivated vaccines. Note: According to Chi-square analysis, the percentage of cases with the NTAb titer changes ≤4 fold difference among all six groups was not significantly different (χ^2^ = 2.04, p>0.05). Comparison of the post-vaccination sera of initially seronegative and seropositive infants and children found no significant differences (χ2 = 1.89, p>0.05). In addition, comparison between vaccine A and vaccine B revealed no significant differences in their EV71 NTAb titers (χ2 = 1.26, p>0.05).

In this part of study, to analyze the serum cross-neutralizing capacity of participants immunized with C4 vaccines, the sera of initially seronegative participants and initially seropositive participants were tested with 5 different genotypes strains and compared with data of sera from subclinically infected participants and clinically infected patients. The fold difference of each serum sample was calculated as highest NTAb titer/lowest NTAb titer among 5 EV71 subgenotypes. The fold difference was used to analyze the cross-neutralizing capacity of sera with different types, which was a common index for evaluating the cross-neutralizing capacity of sera [35], [36]. As Figure 2 shown, in the initially seronegative group, there are 24 out of 24 cases showing less than 4-fold differences for vaccine A, and 25 out of 29 cases showing less than 4-fold differences for vaccine B. In the initially seropositive group, there are 22 out of 23 cases showing less than 4-fold differences for vaccine A, and 30 out of 30 cases showing less than 4-fold differences for vaccine B. Forty one out of 43 subclinically infected infants and children showed less than 4-fold difference. Eight out of 8 clinically infected patients showed less than 4-fold differences. Chi-square analysis showed that the percentage of cases with NTAb titer changes ≤4 fold was not significantly varied among above six groups (χ^2^ = 2.04, p>0.05). Four-fold difference rate for the post-vaccination sera was found no significant difference between initially seronegative and seropositive infants and children (χ2 = 1.89, p>0.05). In addition, 4-fold difference rate between vaccine A and vaccine B was found no significant difference in their EV71 NTAb titers (χ2 = 1.26, p>0.05). These results demonstrated that regardless of whether the baseline NTAb titer is positive or negative (NTAb titer >8, or <8 before vaccination), the cross-neutralizing capacity of serum EV71 NTAb induced by either of the two C4 genotype EV71 vaccines is similar to that of the serum EV71 NTAb induced by natural infection. Furthermore, our results demonstrated that both vaccines elicited notable and broad cross-neutralizing activity against five EV71 strain genotypes.

### 4. Comparison of the results obtained from the CPE and ELISpot assays

**Figure 3:**
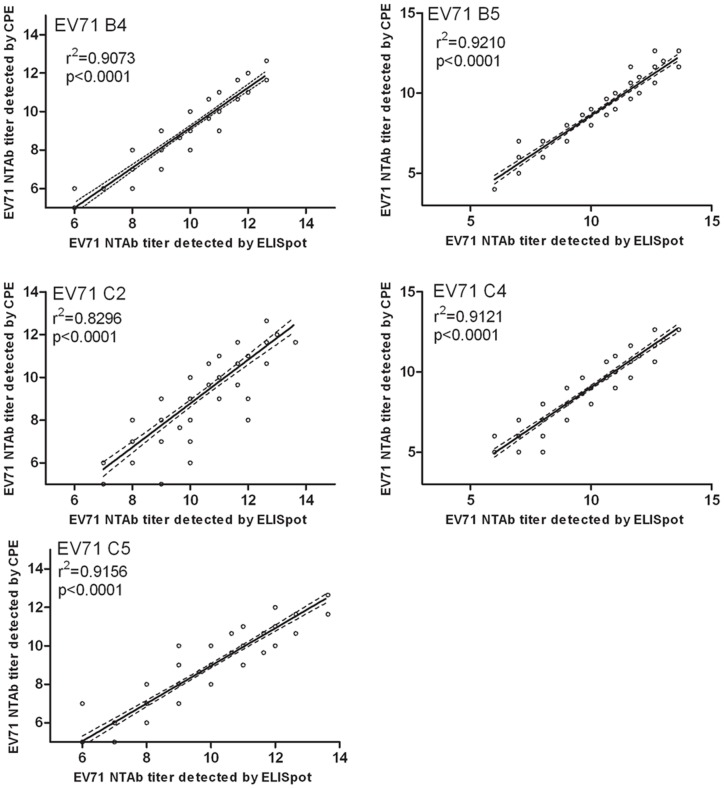
Correlation analyses of the NTAb titers against B4, B5, C2, C4 and C5 by CPE and ELISpot assays. Note: The NTAb titers against B4, B5, C2, C4, and C5, quantified by CPE assays, showed a statistically significant correlation with those detected by ELISopt assays (r^2^≥0.8296, p<0.0001 in both cases).

In order to verify the accuracy of the CPE results, we used China's national NTAb standard as a quality control in each CPE assay ^23^, and ELISpot, a high-throughput detection method, to measure NTAb titers against different EV71 genotypes in all post-vaccination serum samples. The correlation analysis was performed for all samples tested by both the ELISpot and CPE methods with EV71 B4, B5, C2, C4 and C5. The results shown that higher NTAb titer were found in ELISpot assay than in CPE assay for every subgenotype EV71. However, the r^2^ between two assay were 0.9037, 0.9210, 0.8296, 0.9121 and 0.9156 against B4, B5, C2, C4 and C5 subgenotypes, respectively. There were significant relativity between the two assays for each subgenotype EV71(p<0.0001 in both cases; Figure 3). The results demonstrated that there was a good relativity between the two assays, which support the reliablity of the results in this study.

## Discussion

EV71 belongs to *Picornaviridae*, a family of virus that is prone to genetic mutations. Recombinations can occur among EV71 strains of different genotypes, and occasionally even between EV71 and other enterovirus, such as CA16. These events lead to constant changes in the predominant strain in EV71 epidemics [18]. Genotype C2 was the predominant strain of the large HFMD outbreak in Taiwan in 1998 [6]. After 2000, B4 and B5 gradually replaced C2 and C3 as the predominant strains in Taiwan [28], [29]. In Malaysia, EV71 strains C1, C2, B3 and B4, with B3 being the most prominent strain, were detected in 1997 [30]. Between 2000 and 2003, B4 and B5 replaced B3 to become the predominant strains [31]; all of which were then gradually replaced by C1. Epidemiological data from Singapore, Japan and additional regions have all confirmed this trend [32]. However, in China, ever since the first EV71 strain was isolated in 1997, C4 has remained to be the predominant strain [3].

A study of the evolutionary genetics of predominant EV71 strains in the past 40 years demonstrated that the phylogeny of EV71 is characterized by temporal structure with continuous strain replacement over time. This characteristic renders EV71 different from other enteroviruses, notably poliovirus, but similar to human influenza virus, which is a classic example of an antigenically variable pathogen [18]. It remains unclear whether genetic mutations of EV71 lead to alterations in antigenicity like those seen in influenza virus. Presently, little is known about the effect of genotype change on the antigenicity of EV71 [18]. Whether the immune response induced by one vaccine strain can effectively and adequately neutralize other strains is another key factor to consider for evaluating the safety and immunogenicity of these vaccines. To date, EV71 vaccines developed by mainland China, Chinese Taiwan, and Singapore are all vaccines of a single genotype. Thus, studying the cross-protective activity is of great importance for these vaccines.

Some studies have focused on the cross-neutralizing of animal immunized sera,the data demonstrated certain cross-protective activity against different EV71 strains. However, the cross-protection capacity in these animal studies shown more difference and were confusing [33], [34]. And no study has focused on the cross-neutralizing activity of vaccinated human sera, especially those of infants and children, who are the target population, yet such study, could be particularly important for the understanding of the cross-neutralizing immunity elicited by vaccines composed of a single genotype of EV71 strain. In our study, the serum samples from a subset of 119 participants, aged 6 months to 11 years, in two C4 genotype EV71 vaccine clinical trials were used to detect NTAb against EV71 subgenotypes B4, B5, C2, C4 and C5. In order to validate the accuracy of the CPE results, ELISpot was employed in parallel to detect NTAb in all the post-vaccine sera. In both clinical trials, no significant EV71 epidemic was observed at the trial sites; and the EV71 NTAb titer of placebo groups, which were inoculated with aluminium adjuvant, showed no significant increase during the study period [21], [22].

These results showed that, irrespective of the baseline NTAb titres being negative or positive before vaccination, the sero-antibodies of infants and children, who were vaccinated with C4-genotype EV71 vaccines A or B, have broad cross-neutralizing activity against EV71 strains C2, C4, C5, B4 and B5. Forty nine out of 53 participants in initially seronegative group and 52 out of 53 participants in initially seropositive group showed less than 4-fold difference in neutralizing titer after two doses of vaccination, which are close to pediatric patients recovering from EV71-induced HFMD and subclinical-infected infants and children. We further examined paired serum samples from 83 healthy participants before and after vaccination. Results from these samples showed that GMTs of participants against different subgenotypes of EV71 grew significantly after vaccination, irrespective of the baseline NTAb titer being negative or positive. The NTAb-FI against B4, B5, C2, and C5 showed positive correlation to NTAb-FI against C4 with p values. It is surprised and comforted that better cross-protection capacity and smaller neutralizing-antibody differences of human sera were shown than that of animal sera against EV71 with different subgenotype [33], [34].

This study used 43 serum samples of subclinically infected infants and children and 8 serum samples of pediatric patients recovering from EV71-induced HFMD as controls. These samples and the serum samples of vaccinated participants were examined for cross-protective activity against different EV71 strains. The results showed that more than 95.3% serum samples of infected infants and children elicited homogenous cross-protective activity. These results agree with those reported by Mizuta and Huang [35], [36]. Mizuta, *et al.* analyzed the cross-neutralizing ability of sera from 83 residents aged 1 to 60+ years in Japan. Their study suggested differences in the serum NTAb titer of residents against six subgenogroups (B2, B4, B5, C1, C2, and C4) were mostly within 4-fold [35]. Huang, *et al.* examined the cross-neutralizing ability of sera from 25 young Taiwanese children, who were infected by EV71 genotype C2, B4, C4, B5, or C4 from 1998–2010. Their study reported that differences in the serum NTAb infected by genotype C EV71 against all different EV71 subgenotypes, except genotype A, which is the first EV71 genotpye discovered in 1969, were mostly within 4-fold [36]. These results indicated that upon natural infection with EV71 genotype C, humans are able to generate serum antibody with high cross-protective activity against EV71 strains. At present, a highly conserved epitope of VP1, containing amino acids 211–224 (FGEHKQEKDLEYGAC), in the GH loop of the VP1 protein, was confirmed as the dominant linear neutralizing epitope [37]. This epitope was also present in three EV71 vaccine strains of subgenotype C4 and five EV71 detection strains in this study. These results suggesting that although the EV71 genotype defined based on the VP1 sequence was changeable, the neutralizing sites, which perhaps significantly affect the protective activity in humans, could be highly conserved. Evasion from the immune response due to antigenic shift therefore, may not be the main cause of periodic EV71 epidemics. In contrast, newborn accumulation in the epidemic regions, and a decrease of antibody levels in the population, could be the major causes for large-scale EV71 epidemics. This finding is consistent with the results of an epidemiological study conducted in Taiwan that found the positive rate of EV71 NTAb in the examined population was inversely correlated with the incidence of severe EV71 cases and EV71-associated mortality [24].

In this study, EV71strains of genotype B4, B5, C2, C4 and C5 were selected to evaluate the cross-neutralizing effect of serum samples. Genotype C2 and C4 were the predominant strain of the wide HFMD outbreak in Taiwan in 1998 and in mainland china, which also played important role in EV71 epidemic [36], [38]; while B4, B5 and C5 were the prominent strain in the worldwide epidemic over the past decade [31], [39]. Evaluation study of the cross-protective ability of EV71 vaccine using these five genotypes of EV71 is thus more representative, and has better accordance with the EV71 epidemic situation. However, some researchers reported that current genotyping of EV71 does not reflect their antigenicity [40]. For instance, the EV71 C2-like genotype is a special and new subgenogroup of EV71 that was identified in Taiwan in 2008. EV71 C2-like genotype showed significant differences in antigenicity compared to other genotypes EV71 [41]. Therefore, further investigation is needed to identify the cause of the antigenicity change in this specific strain or subgenotype.

In the present study, we first used sera from healthy infants and children to assay the cross-protective activity of two EV71 C4 inactivated vaccines. These data indicated that EV71 vaccination of subgenotype C4 provided the broad and homogeneous protection against different subgenotypes of EV71 in health children and infants, similar to the outcome of natural infection. Altogether, our study demonstrated that the two EV71 inactivated vaccines produced from two different C4 strains, using different manufacturing processes, showed similar cross-protective activity, thereby providing the evidence supporting the broad immunoprotection of EV71- inactivated vaccines of C4 genotype. As newly designed vaccines, the degree of cross-protective immunity elicited, as well as the potential for EV71 to evolve to escape neutralization, remain to be investigated in clinical trials and monitored after market release. Further study of EV71 antigenicity and genetic evolution is warranted. Moreover, when the EV71 vaccine is inoculated simultaneously or at short-intervals with other vaccines, mutual interference or enhancement among them is worth exploring.

## Supporting Information

Table S1 **List of the eight HFMD patients infected with EV71 in Guangxi Province from 2010–2011.**
(DOCX)

Figure S1 **A Phylogenetic tree of EV71 virus strains of different subgenotypes.** Red spots indicate EV71 strains that were used to detect EV71 NTAb in CPE and ELISpot assays of this study.
(TIF)

## References

[1] AbuBakar S, Chan YF, Lam SK (2000) Outbreaks of enterovirus 71 infection. N Engl J Med 342: 355–356. 10660400 10.1056/NEJM200002033420513

[2] Qiu J (2008) Enterovirus 71 infection: a new threat to global public health? Lancet Neurol 7: 868–869. 18848307 10.1016/S1474-4422(08)70207-2 PMC7128195

[3] Solomon T, Lewthwaite P, Perera D, Cardosa MJ, McMinn P, et al (2010) Virology, epidemiology, pathogenesis, and control of enterovirus 71. Lancet Infect Dis 10 11: 778–90 10.1016/S1473-3099(10)70194-8 20961813

[4] Ooi MH, Wong SC, Lewthwaite P, Cardosa MJ, Solomon T (2010) Clinical features, diagnosis, and management of enterovirus 71. Lancet Neurol 9: 1097–1105. 20965438 10.1016/S1474-4422(10)70209-X

[5] Hsu CH, Lu CY, Shao PL, Lee PI, Kao CL, et al (2011) Epidemiologic and clinical features of nonpolio enteroviral infections in northern Taiwan in 2008. J Microbiol Immunol Infect 44: 265–273. 21524954 10.1016/j.jmii.2011.01.029

[6] Ho M, Chen ER, Hsu KH, Twu SJ, Chen KT, et al (1999) An epidemic of enterovirus 71 infection in Taiwan. Taiwan Enterovirus Epidemic Working Group. N Engl J Med 341: 929–935. 10498487 10.1056/NEJM199909233411301

[7] (2008) Notice. The hand-foot-mouth disease integrates the legal infectious disease to manage. Available at: http://www.gov.cn/gzdt/2008-05/03/ content_960344.htm. Accessed May 3, 2008.

[8] McMinn P, Lindsay K, Perera D, Chan HM, Chan KP, et al (2001) Phylogenetic analysis of enterovirus 71 strains isolated during linked epidemics in Malaysia, Singapore, and Western Australia. J Virol 75: 7732–7738. 11462047 10.1128/JVI.75.16.7732-7738.2001 PMC115010

[9] Lee MS, Chang LY (2010) Development of enterovirus 71 vaccines. Expert Rev Vaccines 9: 149–56 PMID:20109026; 10.1586/erv.09.152 20109026

[10] Bek EJ, Hussain KM, Phuektes P, Kok CC, Gao Q, et al (2011) Formalin-inactivated vaccine provokes cross-protective immunity in a mouse model of human enterovirus 71 infection. Vaccine 29 29–30: 4829–38. 21550375 10.1016/j.vaccine.2011.04.070

[11] Zhu FC, Liang ZL, Li XL, Ge HM, Meng FY, et al (2013) Immunogenicity and safety of an enterovirus 71 vaccine in healthy Chinese children and infants: a randomised, double-blind, placebo-controlled phase 2 clinical trial. Lancet 381: 1037–45 doi:pii: S0140-6736(12)61764-4 23352749 10.1016/S0140-6736(12)61764-4

[12] Liang Y, Zhou X, Yang E, Pu J, Che Y, et al (2012) Analysis of the Th1/Th2 reaction in the immune response induced by EV71 inactivated vaccine in neonatal rhesus monkeys. J Clin Immunol 32 5: 1048–58. 22585051 10.1007/s10875-012-9690-3

[13] Chou AH, Liu CC, Chang CP, Guo MS, Hsieh SY, et al (2012) Pilot scale production of highly efficacious and stable enterovirus 71 vaccine candidates. PLoS One 7 4: e34834. 22529942 10.1371/journal.pone.0034834 PMC3328501

[14] Chong P, Hsieh SY, Liu CC, Chou AH, Chang JY, et al (2012) Production of EV71 vaccine candidates. Hum Vaccin Immunother 8 12: 1775–1783. 22992566 10.4161/hv.21739 PMC3656065

[15] Mao Q, Dong C, Li X, Gao Q, Guo Z, et al (2012) Comparative analysis of the immunogenicity and protective effects of inactivated EV71 vaccines in mice. PLoS One 7 9: e46043. 23029378 10.1371/journal.pone.0046043 PMC3460965

[16] Brown BA, Oberste MS, Alexander JP Jr, Kennett ML, Pallansch MA (1999) Molecular epidemiology and evolution of enterovirus 71 strains isolated from 1970 to 1998. J Virol 73: 9969–75. 10559310 10.1128/jvi.73.12.9969-9975.1999 PMC113047

[17] Tu PV, Thao NTT, Perera D, Khanh TH, Tien NTK, et al (2007) Epidemiologic and virologic Invest. of hand, food, and mouth disease, southern Vietnam, 2005. Emerg Infect Dis 13: 1733–1741. 18217559 10.3201/eid1311.070632 PMC3375788

[18] Tee KK, Lam TT, Chan YF, Bible JM, Kamarulzaman A, et al (2010) Evolutionary genetics of human enterovirus 71: origin, population dynamics, natural selection, and seasonal periodicity of the VP1 gene. J Virol 84 7: 3339–50 10.1128/JVI.01019-09 20089660 PMC2838098

[19] Bible JM, Pantelidis P, Chan PK, Tong CY (2007) Genetic evolution of enterovirus 71: epidemiological and pathological implications. Rev Med Virol 17 6: 371–9. 17487831 10.1002/rmv.538

[20] WHO (2011) A Guide to clinical management and public health response for hand, foot and mouth disease (HFMD). http://www.wpro.who.int/publications/docs/GuidancefortheclinicalmanagementofHFMD.pdf (accessed Jan 25, 2013).

[21] Zhu FC, Wang JZ, Li XL, Liang ZL, Ge HM, et al (2012) Reactogenicity and immunogenicity of an enterovirus 71 vaccine in chinese healthy children and infants. Pediatr Infect Dis J 31 11: 1158–65 10.1097/INF.0b013e31826eba74 22926209

[22] Li YP, Liang ZL, Gao Q, Huang LR, Mao QY, et al (2012) Safety and immunogenicity of a novel human Enterovirus 71 (EV71) vaccine: a randomized, placebo-controlled, double-blind, Phase I clinical trial. Vaccine 30 22: 3295–303 10.1016/j.vaccine.2012.03.010 22426327

[23] Liang ZL, Mao QY, Gao Q, Li XL, Dong CH, et al (2011) Establishing China's national standards of antigen content and neutralizing antibody responses for evaluation of enterovirus 71 (EV71) vaccines. Vaccine 29: 9668–9674. 22015395 10.1016/j.vaccine.2011.10.018

[24] Chang LY, King CC, Hsu KH, Ning HC, Tsao KC, et al (2002) Risk factors of enterovirus 71 infection and associated hand, foot, and mouth disease/herpangina in children during an epidemic in Taiwan. Pediatrics 109 6: e88. 12042582 10.1542/peds.109.6.e88

[25] Zielinska E, Liu D, Wu HY, Quiroz J, Rappaport R, et al (2005) Development of an improved microneutralization assay for respiratory syncytial virus by automated plaque counting using imaging analysis. Virol J 2: 84. 16281972 10.1186/1743-422X-2-84 PMC1308871

[26] Abai AM, Smith LR, Wloch MK (2007) Novel microneutralization assay for HCMV using automated data collection and analysis. J Immunol Methods 322: 82–93. 17343873 10.1016/j.jim.2007.02.001 PMC1933494

[27] Cheng T, He DL, Cai YJ, Yang LS, Li ZQ, et al (2013) A novel and rapid elispot assay measuring neutralizing antibody against enterovirus 71. J Virol Methods in press.

[28] Wang JR, Tuan YC, Tsai HP, Yan JJ, Liu CC, et al (2002) Change of major genotype of enterovirus 71 in outbreaks of hand-foot-and-mouth disease in Taiwan between 1998 and 2000. J Clin Microbiol 40: 10–5. 11773085 10.1128/JCM.40.1.10-15.2002 PMC120096

[29] Huang SW, Hsu YW, Smith DJ, Kiang D, Tsai HP, et al (2009) Reemergence of enterovirus 71 in 2008 in Taiwan: dynamics of genetic and antigenic evolution from 1998 to 2008. J Clin Microbiol 47: 3653–62. 19776232 10.1128/JCM.00630-09 PMC2772620

[30] AbuBakar S, Chee HY, Al-Kobaisi MF, Xiaoshan J, Chua KB, et al (1999) Identification of enterovirus 71 isolates from an outbreak of hand, foot and mouth disease (HFMD) with fatal cases of encephalomyelitis in Malaysia. Virus Res 61: 1–9. 10426204 10.1016/s0168-1702(99)00019-2

[31] Podin Y, Gias EL, Ong F, Leong YW, Yee SF, et al (2006) Sentinel surveillance for human enterovirus 71 in Sarawak, Malaysia: lessons from the first 7 years. BMC Public Health 6: 180. 16827926 10.1186/1471-2458-6-180 PMC1543637

[32] Huang SW, Kiang D, Smith DJ, Wang JR (2011) Evolution of re-emergent virus and its impact on enterovirus 71 epidemics. Exp Biol Med (Maywood) 236 8: 899–908 10.1258/ebm.2010.010233 21715436

[33] van der Sanden S, van der Avoort H, Lemey P, Uslu G, Koopmans M (2010) Evolutionary trajectory of the VP1 gene of human enterovirus 71 genogroup B and C viruses. J Gen Virol 91: 1949–1958. 20375223 10.1099/vir.0.019695-0

[34] Arita M, Nagata N, Iwata N, Ami Y, Suzaki Y, et al (2007) An attenuated S of enterovirus 71 belonging to genotype a showed a broad spectrum of antigenicity with attenuated neurovirulence in cynomolgus monkeys. J Virol 81: 9386–9395. 17567701 10.1128/JVI.02856-06 PMC1951441

[35] Mizuta K, Aoki Y, Suto A, Ootani K, Katsushima N, et al (2009) Cross-antigenicity among EV71 strains from different genogroups isolated in Yamagata, Japan, between 1990 and 2007. Vaccine 27: 3153–3158. 19446185 10.1016/j.vaccine.2009.03.060

[36] Huang ML, Chiang PS, Chia MY, Luo ST, Chang LY, et al (2013) Cross-reactive Neutralizing Antibody Responses to Enterovirus 71 Infections in Young Children: Implications for Vaccine Development. PLoS Negl Trop Dis 7 2: e2067 10.1371/journal.pntd.0002067 23459633 PMC3573098

[37] Ku Z, Ye X, Huang X, Cai Y, Liu Q, et al (2013) Neutralizing Antibodies Induced by Recombinant Virus-Like Particles of Enterovirus 71 Genotype C4 Inhibit Infection at Pre- and Post-attachment Steps. PLoS One 8 2: e57601 10.1371/journal.pone.0057601 23451250 PMC3579802

[38] Zhang Y, Tan X, Cui A, Mao N, Xu S, et al (2013) Complete genome analysis of the c4 subgenotype strains of enterovirus 71: predominant recombination c4 viruses persistently circulating in china for 14 years. PLoS One 8 2: e56341 10.1371/journal.pone.0056341 23441179 PMC3575343

[39] Tu PV, Thao NT, Perera D, Huu TK, Tien NT, et al (2007) Epidemiologic and virologic investigation of hand, foot, and mouth disease, southern Vietnam, 2005. Emerg Infect Dis 13: 1733–41. 18217559 10.3201/eid1311.070632 PMC3375788

[40] Chen Y, Li C, He D, Cheng T, Ge S, et al (2013) Antigenic analysis of divergent genotypes human Enterovirus 71 viruses by a panel of neutralizing monoclonal antibodies: current genotyping of EV71 does not reflect their antigenicity. Vaccine 31 2: 425–30 10.1016/j.vaccine.2012.10.032 23088887

[41] Huang YP, Lin TL, Hsu LC, Chen YJ, Tseng YH, et al (2010) Genetic diversity and C2-like subgenogroup strains of enterovirus 71, Taiwan, 2008. Virol J 7: 277 10.1186/1743-422X-7-277 20959020 PMC2975644

