# Supplementary material for: The Cross-Neutralizing Activity of Enterovirus 71 Subgenotype C4 Vaccines in Healthy Chinese Infants and Children
Source: PLoS One. 2013 Nov 19;8(11):e79599. doi: 10.1371/journal.pone.0079599 (PMC3834186; doi:10.1371/journal.pone.0079599)
Supplement: Table S1 — List of the eight HFMD patients infected with EV71 in Guangxi Province from 2010–2011. (DOCX) [file pone.0079599.s001.docx]

**Table S1.** List of the eight HFMD patients infected with EV71 in Guangxi Province from 2010-2011.

| NO. | Gender | Age  (months) | Symptoms | Diagnosis | EV71 RNA |
| --- | --- | --- | --- | --- | --- |
| PA01 | F | 40 | Fever (38.5°C), skin rash (hands, feet and hip), and coughing | HFMD | + |
| PA02 | F | 35 | Fever (38.2°C), skin rash (hands and feet), stomatitis, and coughing | HFMD | + |
| PA03 | F | 43 | Skin rash (hands, feet and hip), stomatitis, coughing, and runny nose | HFMD | + |
| PA04 | M | 20 | Fever (42.0°C), skin rash (hands and feet), stomatitis, and coughing | HFMD | + |
| PA05 | F | 53 | Fever (39.0°C), stomatitis, and runny nose | HFMD, acute suppurative tonsillitis | + |
| PA06 | M | 40 | Fever (38.0°C), skin rash (hands and feet), and stomatitis | HFMD | + |
| PA07 | M | 30 | Fever (37.5°C), skin rash (hands, feet and hip), and stomatitis | HFMD | + |
| PA08 | F | 73 | Fever (39.5°C) and stomatitis | HFMD | + |
