# Supplementary figures and images for: The Cross-Neutralizing Activity of Enterovirus 71 Subgenotype C4 Vaccines in Healthy Chinese Infants and Children
Source: PLoS One. 2013 Nov 19;8(11):e79599. doi: 10.1371/journal.pone.0079599 (PMC3834186; doi:10.1371/journal.pone.0079599)

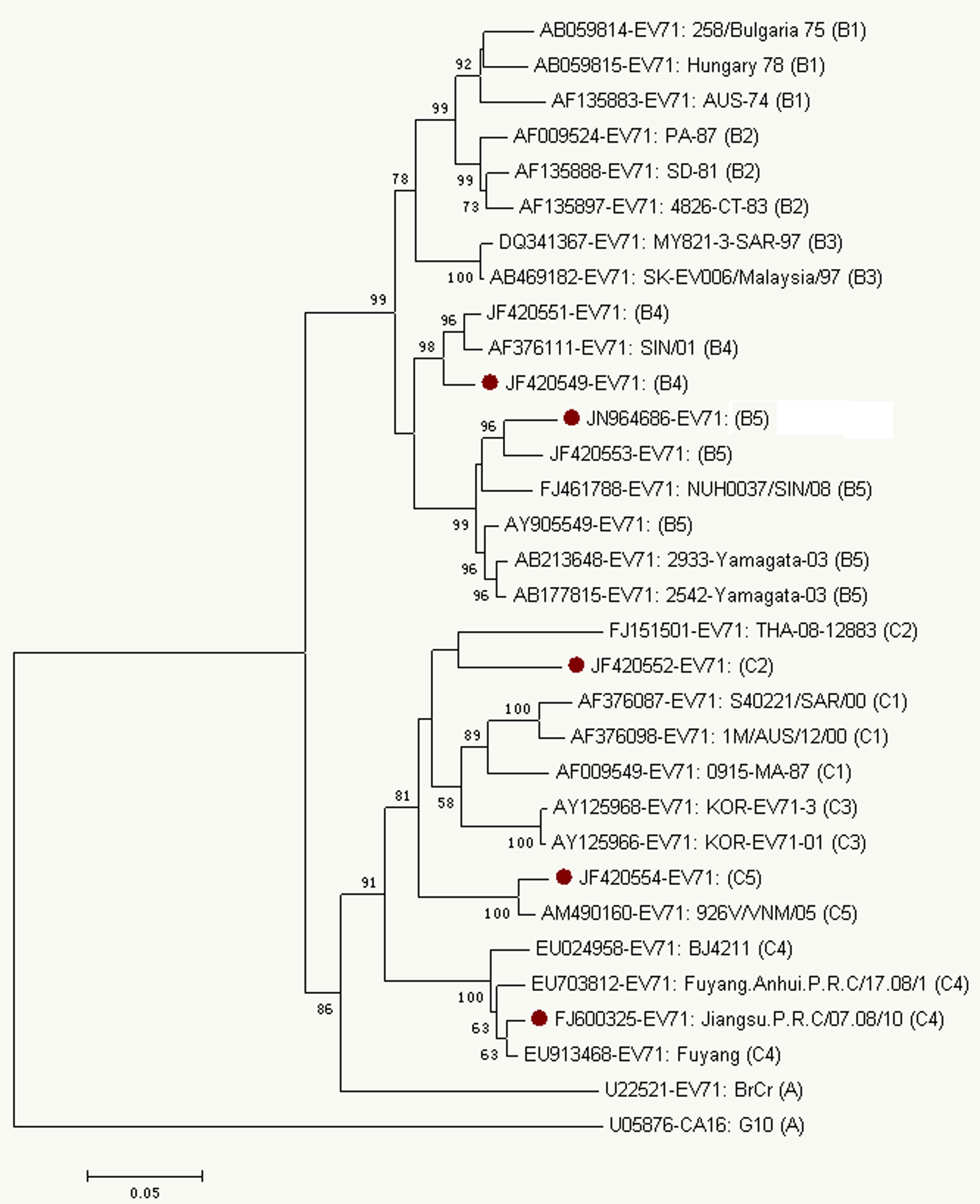

Supplement: Figure S1 — A Phylogenetic tree of EV71 virus strains of different subgenotypes. Red spots indicate EV71 strains that were used to detect EV71 NTAb in CPE and ELISpot assays of this study. (TIF) [file pone.0079599.s002.tif]
